# AntibioticDB: An Updated and Improved Open-Access Database for the Antibacterial Research and Development Community

**DOI:** 10.1021/acsinfecdis.5c00955

**Published:** 2026-02-23

**Authors:** Luiza H. Galarion, Alan Hennessy, Simon D. Harding, Jane F. Armstrong, Astrid Pentz-Murr, Jamie A. Davies, Alex J. O’Neill, Laura J. V. Piddock

**Affiliations:** † Faculty of Biological Sciences, School of Molecular and Cellular Biology, 4468 University of Leeds, Leeds LS2 9JT, U.K.; ‡ 542454 The Global Antibiotic Research & Development Partnership (GARDP), Geneva 1202, Switzerland; § Institute for Neuroscience and Cardiovascular Research, 3124 University of Edinburgh, Edinburgh EH8 9XD, U.K.; ∥ Edinburgh Medical School, University of Edinburgh, Edinburgh EH8 9XD, U.K.

**Keywords:** AntibioticDB, database, antibiotic, antibacterial, structure, chemical

## Abstract

AntibioticDB (https://www.antibioticdb.com/), originally established in 2017 and since 2021 led by the Global Antibiotic Research & Development Partnership (GARDP), is a freely available database of antibacterial agents to facilitate research and development of new antibacterial therapeutics. Here, we describe a new release of AntibioticDB that has been significantly expanded and updated with the aid of user feedback and which offers additional functionality through a redesigned web portal. Improvements include reciprocal integration with the IUPHAR/BPS Guide to Pharmacology (https://www.guidetopharmacology.org), capturing of compound structure information in the form of standard chemical identifiers (canonical and isomeric SMILES, InChI, and InChI Key), chemical 2D structure images, and harmonizing terminology to optimize database searching. Ongoing curation efforts have increased the number of individual entries to >3,500, a process driven mostly by a significant expansion of historical natural product antibiotics that were previously under-represented in the database. The database is continuously updated by mining the published literature and capturing newly discovered antibacterial compounds as they are reported, making AntibioticDB the most complete global resource on antibacterial agents.

## Graphical abstract

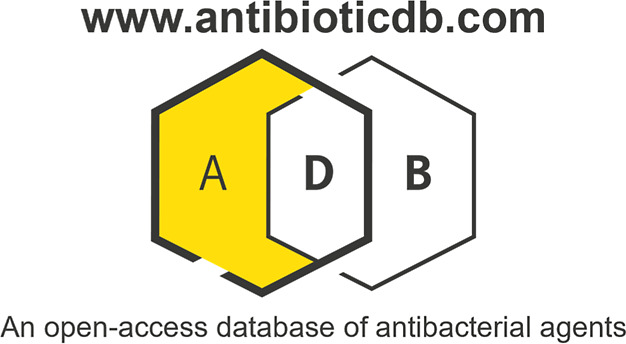

Antimicrobial resistance (AMR) is a global public health issue, of which the considerable scale has only recently begun to be clearly defined. The Global Burden of AMR Study estimated that in a single year (2019), 1.27 million deaths worldwide were attributable to drug-resistant bacterial infections, with a further 4.95 million deaths associated with AMR.^1^ The latest forecasts suggest that, by 2050, these figures will have risen to 1.91 million attributable deaths and 8.22 million associated deaths.^2^ Addressing the challenge of AMR requires action on multiple fronts, including improving infection prevention and control to reduce the use of antimicrobials, along with enhanced resistance surveillance and stewardship to optimize antimicrobial use.^3^ While such approaches should serve to limit the rate at which the AMR problem escalates, it nonetheless remains essential to develop new antibacterial treatments effective against pathogens that have evolved resistance to existing antibiotics. Unfortunately, this is easier said than done—the antibacterial pipeline remains extremely underpopulated for several reasons that are well recognized.^4,5^

One of the most fundamental issues in bringing new antibacterial drug candidates forward is identifying suitable agents to develop. Revisiting drug candidates whose development was originally discontinued presents one potential approach to revitalizing the antibacterial pipeline. Indeed, rehabilitating failed antibacterials to deliver new drug candidates represents a tantalizing yet realistic prospect; the field has witnessed several instances where antibacterial compounds were initially discarded, but which were subsequently revisited and developed into successful antibacterial drugs (e.g., daptomycin,^6^ fidaxomicin^7^). This was the original motivation behind AntibioticDB (https://antibioticdb.com), an online, freely- and globally-accessible database that was established in 2017 to capture detailed information regarding agents that have—or that may possess—potential for use in antibacterial chemotherapy.^8^

It is already apparent that AntibioticDB is proving a valuable resource for the research and drug discovery community; the publication describing it^8^ has been cited >60 times and, in the period April 1, 2018 to October 28, 2021, 4025 users accessed the database a total of 17,690 times (*unpublished data*). From 2021, the expansion and further evolution of AntibioticDB have been supported and led by the Global Antibiotic Research & Development Partnership (GARDP). Curation and database/website development are being undertaken, respectively, by researchers from the University of Leeds (LG, AJO) and the University of Edinburgh (SH, JA and JD). Here, we provide an overview of the new release of the updated AntibioticDB and describe the improvements in content and functionality that have been achieved since the initial description was published in 2018.^8^

## Expanding Database Content

The number of individual entries in the database is now over 3,500, a >4-fold increase over the original release reported in 2018. These new entries have been identified through extensive literature searching and come from diverse sources including those listed by Farrell et al.^8^ Two key sources are worthy of specific mention since they have allowed a dramatic increase in the capture of historical natural product antibiotics in the database: The Journal of Antibiotics (https://www.jstage.jst.go.jp/browse/antibiotics1968/-char/en) and the Encyclopaedia of Antibiotics.^9^ Thousands of such compounds have been described in the scientific literature over the last ∼80 years yet were under-represented in the first iteration of AntibioticDB. Because most antibacterial drugs in clinical use are, or derive from, natural product antibiotics, molecules of this type represent an important likely source of future treatments for bacterial infection.

The process of identifying new entries to add to the AntibioticDB database is guided by the following key principles:

(i) Agents should demonstrate a degree of selective toxicity against bacteria over mammalian cells. This is a defining feature of an agent with potential for use in systemic antibacterial chemotherapy, since it makes feasible specific targeting of disease-causing bacteria without comparable toxic effects on the patient. It is rarely possible to “introduce” selective toxicity by modification of a compound that lacks selectivity at the outset because such agents typically exert their effects on bacterial and mammalian cells through the same mechanism. Thus, compounds lacking selectivity of action have limited value or prospects for antibacterial chemotherapy and are not the focus of AntibioticDB. An “indication of selectivity” can take many forms in the published literature including a demonstration of safety/efficacy in vivo, a difference in the observed response to the compound between cultured bacterial and mammalian cells, or inhibitory action against a purified target protein that is reduced or absent against the mammalian counterpart.
(ii) Historically, the major focus in antibacterial chemotherapy has been on small molecule drugs that inhibit or kill bacteria (“direct-acting antibacterials”), and such molecules also constitute the bulk of entries in AntibioticDB. Nevertheless, there are agents in clinical use that do not act in this manner (e.g., beta-lactamase inhibitors that primarily act to inhibit an antibiotic resistance mechanism), and there is a growing interest in “nontraditional” agents that act through alternative means.^10^ Any modality that has, or might have, potential in the prevention or treatment of bacterial infection is considered appropriate for inclusion in AntibioticDB. Thus, included in AntibioticDB are agents that lack intrinsic antibacterial activity (e.g., those that sensitize bacteria to antibacterial drugs, that target bacterial virulence functions as opposed to viability, or that modulate the host response to infection) and those that are considerably larger than the classical antibacterial drug (e.g., biological materials like enzymes, antibodies, and bacteriophages). Where such agents are intended to be combined with a specific antibacterial drug during treatment, each of the individual components and the combination treatment are curated in AntibioticDB as independent entries.
(iii) Close chemical analogues of antibacterial agents generated during discovery programs are not captured as separate entries in AntibioticDB unless there is a clear rationale for doing so. Often, such “project compounds” are not all individually characterized and/or reported in detail in the published literature, and the assumption that these molecules have the same antibacterial mode of action as the lead compound may not have been verified. Thus, there is typically a lack of robust data available to warrant dedicated entries for such analogues in AntibioticDB. Instead, information is captured for the core or lead scaffold, and the existence of reported analogues is indicated. Exceptions to this approach are made when compelling reported data for an analogue indicate an important difference to other compounds of the series (e.g., distinct antibacterial spectrum or mode of action).

## Improved Information Capture and Harmonization of Terminology in AntibioticDB

In addition to adding database content in the form of new entries, more detailed information is now being captured per entry to maximize utility for the user. To better enable users to search the database for compounds with specific properties, the terminology used to describe antibacterial agents and their activity has also been streamlined and harmonized.

For example, information on the origin of antibacterial agents is now included, showing whether they are natural products (and if so, the producer organism or source), semisynthetic derivatives of natural products, or wholly synthetic compounds synthesized in the laboratory. Where available, additional relevant detail regarding the therapeutic potential of agents has been added (e.g., data on cytotoxicity, key results of in vivo efficacy studies). AntibioticDB has now formalized the incorporation of synonyms for compound names, capturing what we consider to be all relevant terms including abbreviations or acronyms, alternative iterations for alphanumeric names, and brand names for proprietary drugs. The major motivation for this was to improve database searching but has also had the benefit of allowing improved detection and resolution of duplicate entries in the database.

Another improvement in AntibioticDB is a better-defined terminology and a more systematic approach to classifying antibacterial agents. In cases where chemical compounds belong to an established antibiotic or antibacterial drug class (e.g., beta-lactams, tetracyclines), we consider that this is the most appropriate/useful “class” information to display. A comparable approach is used for compounds that fall into other recognized groupings (e.g., “antimicrobial peptide”). Where categorization on this basis is not possible, entries are categorized according to their *functional* class. For example, in the case of direct-acting antibacterials for which the mode of action is known, the “class” will describe the cellular pathway and/or specific drug target inhibited (e.g., fatty acid synthesis inhibitor [FabI inhibitor]). Indirect-acting and nontraditional antibacterial modalities are also referred to by function, using a defined vocabulary (e.g., antibiofilm agent, antivirulence agent). Where agents cannot be classified according to either their class or function, they are described according to their basic nature (e.g., small molecule antibacterial agent).

Where available, entries for drugs will include information on the current status and highest development stage (Table 1). To provide more details in the data captured, the “Highest Development Stage” field now contains the clinical phase of a drug as registered in https://clinicaltrials.gov and their corresponding NCT reference, or an “Approved” status along with the year of first approval by FDA, EMA, or equivalent. The “Development Status” field indicates the current status of an agent using the defined vocabulary described in Table 1. Antibacterial agents that have not progressed to clinical trials, including those in early discovery through to preclinical evaluation, are designated “Experimental”. Agents with the status “Inactive” are those that have been terminated during or after clinical trials, while those designated “Withdrawn” have gained approval but were subsequently withdrawn from clinical use. As part of the ongoing curation, AntibioticDB will maintain updates to the status of current entries when they are in clinical development or approved for use (Figure 1).

**Table 1:** Redefined Terms in the New Release of AntibioticDB

“development status” field term	definition
approved	drugs approved by FDA, EMA, or other drug regulatory agencies and currently available in ≥1 country
active	agents in clinical trials (whether ongoing or completed, with or without results posted)
inactive	agents that have at least reached phase 1 clinical trials and are no longer being taken forward
experimental	agents in discovery or preclinical stages
withdrawn	drugs previously approved but discontinued/withdrawn from market
unknown	agents for which clinical trials have been performed or published but have not been approved for reasons unknown

“highest development stage” field term	definition
preclinical	agents explicitly reported to be undergoing preclinical evaluation (e.g., in a published article, company press release, or by direct communication from researchers)
phase 1–4	agents undergoing clinical trial (reference number included where available)
approved	approved status (with approving agency and year of first approval shown)

**Figure 1:**
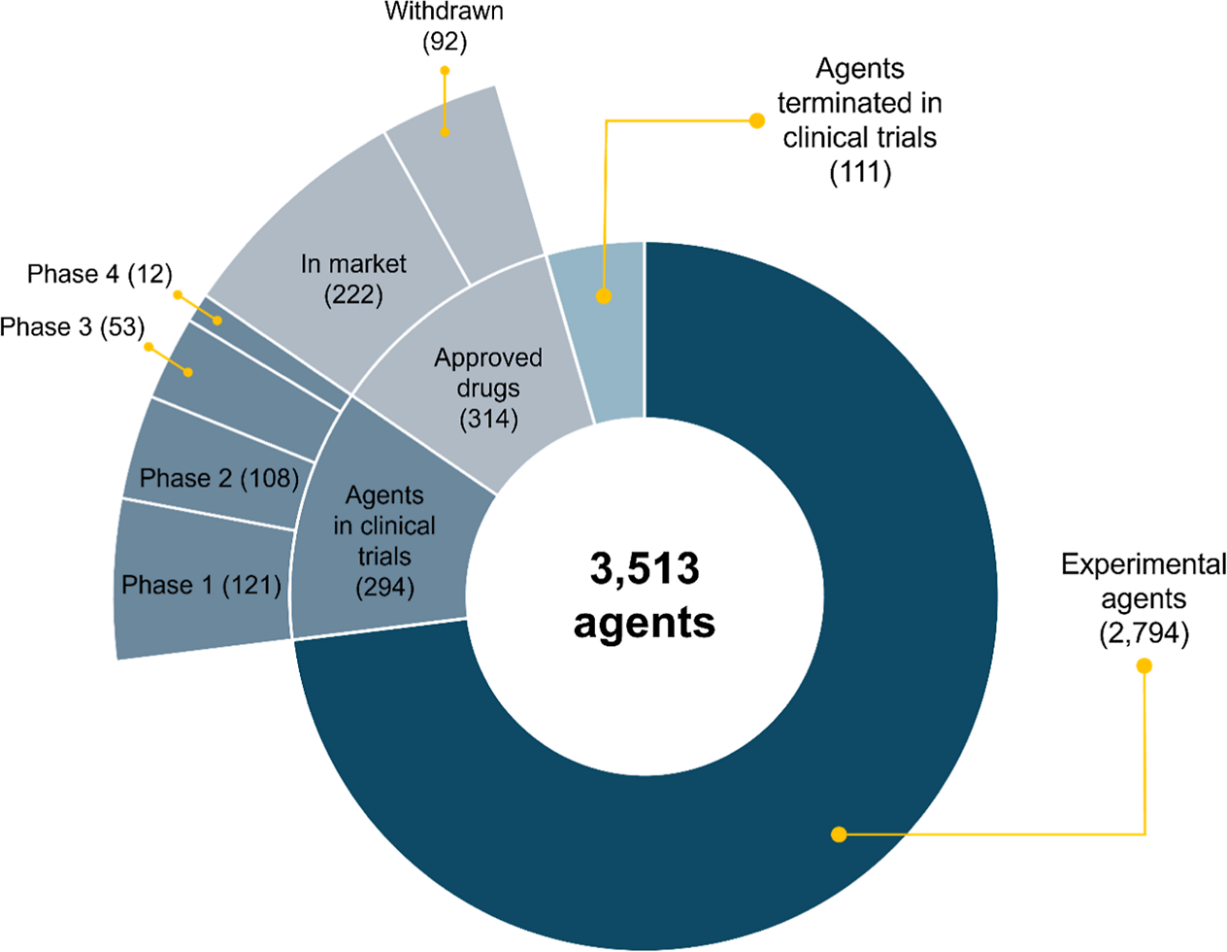
Agents in AntibioticDB as of October 2025 and their development status.

## New Functionality in AntibioticDB

Beginning in 2019, AntibioticDB has benefitted from reciprocal integration with the IUPHAR/BPS Guide to Pharmacology (GtoPdb; https://www.guidetopharmacology.org).^11,12^ GtoPdb is a long-established and widely used database of pharmacological targets and the ligands that act on them and includes drugs for both infectious and noninfectious diseases. The information captured in GtoPdb is distinct from that in AntibioticDB; the latter captures microbiological data and includes antibacterial agents that are at any point along the discovery-development continuum, while the former has a focus on biochemical properties and pharmacodynamics of drugs/advanced drug candidates. Nevertheless, the information in the two databases is highly complementary. To date, ∼19% (687 of 3513) of AntibioticDB entries have a reciprocal GtoPdb link.

From the outset, AntibioticDB has provided external links—where available—to chemical compound information held in PubChem (https://pubchem.ncbi.nlm.nih.gov).^13^ A primary reason for this is to provide users of AntibioticDB access to structural information on chemical compounds. As of October 2025, ∼54% of entries in AntibioticDB have an associated PubChem link. Structural information is now also captured directly in AntibioticDB; not only does this allow medicinal chemists to search AntibioticDB for compounds of interest based on specific chemical features but it also offers researchers structural information for antibacterial compounds that lack a PubChem entry. Thus, the updated AntibioticDB captures this information in the form of standard chemical identifiers, using several common formats (canonical SMILES, isomeric SMILES, InChI, and InChI Key). The process used to do this involves first identifying (or generating) an isomeric SMILES string. For many compounds, this information can be extracted from PubChem or other open-access chemical databases (ChEMBL^14^ or Japan Chemical Substance Dictionary [http://doi.org/10.15079/NIKKAJI]). For compounds that do not have a reported chemical identifier, a structure from the original report of the compound is submitted to either Revvity ChemDraw Prime 23 or DECIMER^15^ (https://decimer.ai/) to generate the SMILES string. To guarantee a uniform format of chemical identifiers, the isomeric SMILES input is then converted by a structure generator (Chemistry Development Kit CDK 2.9—https://cdk.github.io/)^16^ to create other chemical strings and keys (canonical SMILES, InChI, and InChI Key) and a 2D structure depiction with corresponding molecular weight. As of October 2025, ∼67% (2338 of 3513) of entries in AntibioticDB had chemical identifiers. The inclusion of such identifiers has made it possible to incorporate a search functionality based on chemical structure (https://www.antibioticdb.com/chemSearch.jsp), where a query input can either be a SMILES string or drawn using a built-in chemical drawing tool. This facility will now allow users to look for matching substructures or similar structures based on their Tanimoto coefficients, providing a powerful new way to explore compound data in AntibioticDB.

## An Improved Database and Web Portal

An important aspect in the development of the improved AntibioticDB has been the switch from using a JavaScript (Node.js) and Microsoft Excel spreadsheet to using a PostgreSQL relational database. This switch will help to ensure data integrity and consistency. Another advantage of this change is more efficient data retrieval, which provides a better basis for implementing searches and filtering across AntibioticDB, including the ability to filter or download search results. These changes have also helped to link compound data in AntibioticDB more easily to multiple publication sources, patents, and structure links and provide the platform for the curation of chemical structure data.

To enhance the user experience, several improvements have been made to the AntibioticDB website that include reorganization of the structure and information provided on the site. An example of an individual entry page is presented in Figure 2, showing examples of the linked information that is now available. AntibioticDB now incorporates “wild-card” searching, allowing use of partial search terms when they are entered followed by an asterisk; this facilitates searching in several ways, including when looking for groups of related compounds that share a prefix or where variations in spelling exist for a particular agent. Additionally, the search function now features text autocomplete, displaying a drop-down list of agent names matching to a query. When AntibioticDB was originally established, the search function simply returned all occurrences of the query in the database in order of appearance. To improve search performance, the results are now ranked, with the top-ranked hit(s) returned for matches in agent name followed by matches under the heading of agent class. As a rule, a query returning several hits will prioritize entries with more occurrences of the query term. The results returned for any given search can now be downloaded as a comma-separated value (.csv) file for further interrogation offline. Full database content can also be browsed as a list or downloaded in full.

**Figure 2:**
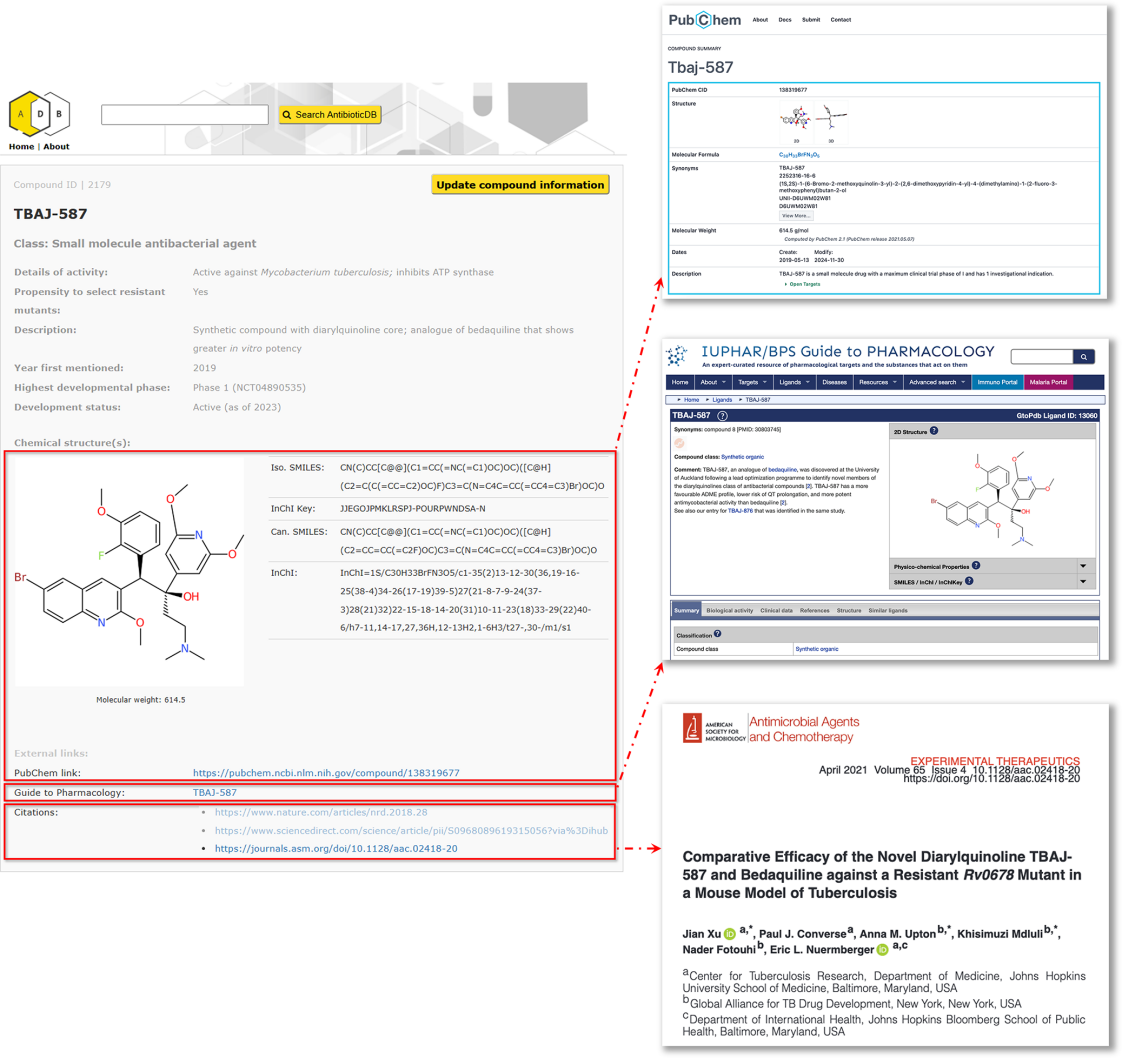
Layout and content of a typical entry page in the new release of AntibioticDB. The entry page is shown in the left-hand panel; linked information is highlighted in red boxes and directs users to relevant information held in other databases.

## Concluding Remarks and Future Directions

AntibioticDB remains the only database of antibacterial agents that is expertly curated and freely available worldwide. As described herein, the database and its web portal have recently undergone considerable development in terms of scope, content, and functionality. This process has been guided and refined by user feedback, and the authors thank the expert users from academia and industry who utilized our beta-testing website during development and provided feedback on their experience (*see* Acknowledgments). Among the key issues raised and successfully addressed in the new release of AntibioticDB are greater capture of historical natural product antibiotics, direct access to structural information, and improved database searching capabilities. The further expansion and evolution of AntibioticDB have significantly increased its value for the antibacterial drug discovery community; in particular, the inclusion of chemical identifiers and a structure-based search functionality now extends its utility to medicinal chemists.

Our current focus for AntibioticDB is on more extensive mining of antibacterial agents from the patent literature and further enhancing database searching capabilities. We encourage the research community to participate in improving AntibioticDB and to send information for inclusion, or feedback, to antibioticdb@gardp.org.
